# Transcriptomic Evidence Reveals the IIS–FOXO–SOD2 Axis as a Core Anti-Aging Pathway in Long-Lived Queens of *Odontotermes formosanus*

**DOI:** 10.3390/insects17040432

**Published:** 2026-04-17

**Authors:** Yiyang Chen, Dandan Qiao, Hao Chen, Dayu Zhang, Yongjian Xie

**Affiliations:** 1 Zhejiang Key Laboratory of Biology and Ecological Regulation of Crop Pathogens and Insect, College of Advanced Agricultural Sciences, Zhejiang A&F University, Hangzhou 311300, China; babel305477179@gmail.com (Y.C.); 15529900209@163.com (D.Q.); zhangdayu@zafu.edu.cn (D.Z.); 2 Jiande Forestry Comprehensive Service Center, Jiande 311699, China; 18480678003@163.com

**Keywords:** differentially expressed gene, gerontology, growth factor, lifespan, longevity, qPCR, social insect, termite

## Abstract

The reproductive caste of higher termites exhibits remarkable longevity, but the mechanisms by which they manage age-related oxidative stress during lifespan extension remain insufficiently understood. This study investigated the dynamic regulation of the insulin/IGF (IIS)–FOXO axis, a key anti-aging regulatory pathway that integrates insulin signaling with downstream processes, including antioxidant defense and DNA repair, as well as the superoxide dismutase (SOD) system in female *Odontotermes formosanus* reproductives at various life stages (Swarming Queen (SQ), 1-Year Queen (1YQ), 8-Year Queen (8YQ)) through transcriptomic, qRT-PCR, and enzyme activity analyses. Age-dependent upregulation of IIS pathway components (*InR*, *chico*, *PDK1*, *Akt*, *Sirt1*, *FOXO*) was observed, alongside the identification of six *SOD* transcripts, including two *SOD1*, two *SOD2*, and two *SOD3* isoforms. Notably, mitochondrial *SOD2* (particularly *SOD2_b*) showed a progressive increase with age, exhibiting the highest enzymatic activity and being associated with reduced mitochondrial oxidative stress and the disruption of reactive oxygen species (ROS) amplification cycles. These findings suggest that *O. formosanus* reproductives counteract the potential lifespan-reducing effects of chronic IIS activation by maintaining or enhancing FOXO activity, thereby supporting DNA repair, antioxidant defenses, and cellular homeostasis. The IIS–FOXO–SOD2 axis is identified as a key regulator of reproductive longevity in higher termites, offering new insights into the molecular mechanisms behind lifespan extension in social insects.

## Simple Summary

Comparative transcriptomic analysis showed that, during queen aging in *Odontotermes formosanus*, the insulin/insulin-like growth factor signaling (IIS) pathway and its downstream regulatory factors are overall persistently upregulated. In aged queens, the elevated expression of *FOXO* may, in concert with its downstream effectors, mitigate the detrimental impact of activated IIS on lifespan extension.

## 1. Introduction

Lifespan extension research has identified various key factors, including immune system function [1], suppression of excessive inflammation [2], autophagy [3], antioxidant activity [4], and DNA repair [5], all of which play pivotal roles in lifespan regulation. The downstream regulation of the IIS pathway integrates these critical processes, establishing it as a central focus in lifespan extension research [6].

The reproductives of higher termites (Termitidae) exhibit an extraordinarily long lifespan, far exceeding that of other insects and social species. Their longevity can surpass 19 years [7], a stark contrast to the relatively short lifespans of reproductives in species like Reticulitermes sp. and bees, which typically live only a few years [8,9]. This remarkable lifespan has driven the evolution of complex mechanisms in higher termites to counteract age-related physiological challenges, such as mitochondrial aging, dysregulation of reactive oxygen species (ROS) signaling [10,11], respiratory chain dysfunction [12], antagonistic pleiotropy gene effects during aging [13,14,15,16], and the accumulation of deleterious mutations [17,18]. These distinctive biological traits position higher termites as a unique model for investigating the mechanisms underlying lifespan extension.

Research in model organisms has shown that suppression of the IIS pathway can significantly extend lifespan [19,20]. However, this regulatory mechanism mainly depends on the key downstream transcription factor of the IIS pathway, forkhead box protein O (FOXO), which links IIS with longevity-promoting mechanisms such as DNA repair and antioxidant defense [21,22].

FOXO, a pivotal transcription factor, regulates several fundamental biological processes, including DNA repair, antioxidant defense, autophagy, and cell cycle control [23]. Among the antioxidant genes regulated by FOXO, manganese superoxide dismutase (MnSOD) is a central component of the primary defense system against oxidative stress, playing a pivotal physiological role [24]. This enzyme is primarily localized in mitochondria, where it mitigates ROS generated during mitochondrial aerobic respiration [25]. MnSOD catalyzes the conversion of highly reactive superoxide radicals (O^2−^) into less harmful hydrogen peroxide (H_2_O_2_), thereby reducing cellular damage caused by ROS [26].

The accumulation of ROS can damage mitochondrial integrity, and dysfunctional mitochondria may further amplify ROS production, initiating a self-perpetuating cycle. This feedback loop contributes to sustained cellular oxidative damage, chronic inflammation, and impaired oxidative phosphorylation, all of which are recognized as major drivers of biological aging [27]. Numerous studies have demonstrated that SOD exerts lifespan-extending effects in various model organisms [28,29,30]. High SOD enzymatic activity has also been observed in an exceptionally long-lived termite [11].

*Odontotermes formosanus* is a significant agricultural and forestry pest in South Asia [31]. The remarkable longevity of its reproductive caste [7] allows mature colonies to reach populations of millions [32], enabling the formation of numerous satellite nests and complicating efforts to control this pest at the root level. Notably, the extended lifespan of the reproductive caste is a key factor in the severe damage caused by *O. formosanus*, making the investigation of their longevity mechanisms both scientifically significant and practically urgent.

Studies on related termites did not clarify the dynamic changes in IIS pathway expression or in the enzymatic activities of its downstream antioxidant enzymes as queens age [3,11].

This study aimed to elucidate the engagement of anti-aging mechanisms during the natural aging process of female reproductives of *O. formosanus*. An age-gradient design was employed to capture age-dependent transcriptional and enzymatic changes within the same caste. Transcriptomic analysis revealed that the expression levels of the IIS pathway and FOXO increased progressively with age. Although activation of the IIS pathway is often thought to suppress anti-aging mechanisms, this theory is based on the premise that activation of the pathway leads to FOXO phosphorylation and consequently impairs its regulatory activity in the nucleus. However, we speculate that if FOXO itself is expressed at a sufficiently high level, it may, as a compensatory effect, help maintain the continuous operation of anti-aging mechanisms in aged individuals even under conditions of activated IIS. By integrating the key transcription factor FOXO and its downstream target genes, this study identified two *SOD1*, two *SOD2*, and two *SOD3* transcripts in *O. formosanus* reproductives. Notably, our findings demonstrated that the enzymatic activity of *SOD2_b* increases progressively with age.

## 2. Materials and Methods

### 2.1. Sample Collection

The 8-year and 1-year *O. formosanus* colonies were maintained and monitored by the Termite Genome and Molecular Laboratory at Zhejiang A&F University. Newly eclosed queen (SQ) samples were collected on 11 May 2024 at the university. Each group consisted of three biological replicates: three SQs, three 1-year queens (1YQs), and three 8-year queens (8YQs). The 1YQs and 8YQs were collected from six different colonies. All samples were stored at −80 °C until further use. To ensure the accuracy of the transcriptome data, RNA was extracted exclusively from the head and thorax tissues of the queens, minimizing interference from the highly abundant metabolic genes in the abdomen.

### 2.2. Sample Processing, Transcriptome Reference Construction, and Transcriptome Sequencing

Long-read cDNA data were used as a reference template, integrated with Illumina-based RNA-seq data. The 8YQ # 1 individual was selected for construction of a reference, as queens are expected to exhibit richer transcriptional profiles [33]. Total RNA was extracted from all tissue samples using the Trizol method (Tiangen DP424) on ice. RNA concentration and purity were assessed prior to library preparation, and only high-quality RNA samples were used for subsequent PacBio and Illumina library construction. Reverse transcription was performed using the PacBio Iso-Seq Express 2.0 kit (Pacific Biosciences, Menlo Park, CA, USA), followed by cDNA purification with SMRtbell magnetic beads for PCR amplification. The Kinnex PCR 8-fold kit (Product #: 103-071-600, PacBio, Menlo Park, CA, USA) was employed to generate DNA fragments containing directional Kinnex segmentation sequences. These cDNA fragments were then ligated to a barcoded Kinnex terminal adapter using the Kinnex ligation kit (Product #: 103-071-800, PacBio, Menlo Park, CA, USA) and linked to the growth fragment. After connecting the stem-loop sequencing adapter and removing failed ligation products, the library template and enzyme complex were loaded into a Revio sequencer (PacBio, Menlo Park, CA, USA) for high-quality full-length cDNA sequencing for transcriptome analysis. Primer sequences were removed using lima (v2.9.0) “https://github.com/PacificBiosciences/barcoding (accessed on 16 August 2025)”. These reads were clustered using the Iso-Seq3 pipeline (https://github.com/ylipacbio/IsoSeq3) to obtain cluster consensus sequences. Additionally, Illumina RNA-seq libraries were constructed from total RNA samples according to the manufacturer’s standard protocol. Briefly, mRNA was enriched using oligo(dT)-attached magnetic beads, fragmented into short pieces, and reverse-transcribed into first-strand cDNA, followed by second-strand cDNA synthesis. After end repair, A-tailing, adapter ligation, and PCR amplification, the qualified libraries were sequenced on the Illumina HiSeq platform to generate 2 × 150 bp paired-end reads (PE150). The cluster consensus sequences were then error-corrected using LoRDEC [34] with the Illumina PE150 reads. Clustered consensus sequences were further clustered using CD-HIT (v4.7) [35] to reduce redundancy. CDS regions were predicted using ANGEL (v2.4) [36].

### 2.3. Annotation of Unigene CDS

Annotation of the CDS sequences was performed using five major databases: KOG “ftp://ftp.ncbi.nih.gov/pub/COG/KOG/kyva (accessed on 17 August 2025)”, NR “http://www.ncbi.nlm.nih.gov (accessed on 17 August 2025)”, GO “https://geneontology.org (accessed on 17 August 2025)”, KEGG “http://www.genome.jp/kegg (accessed on 17 August 2025)”, and SwissProt “http://www.expasy.ch/sprot (accessed on 17 August 2025)”. Annotations for the Clusters of Orthologous Groups (COG) and Gene Ontology (GO) were performed using internal custom scripts, whereas NR, KEGG, and SwissProt annotations were carried out using the blastx function of DIAMOND (v2.1.2) [37]. The annotation results yielded 15,112, 25,969, 11,304, 14,981, and 17,339 matching sequences for KOG, NR, GO, KEGG, and SwissProt, respectively, with annotation rates of 39.27%, 67.48%, 29.37%, 38.39%, and 45.06%.

### 2.4. Differentially Expressed Genes

Illumina PE150 short reads were mapped to the full-length cDNA reference using minimap2 (v 2.30) [38]. Transcript abundance was quantified using StringTie (v 3.0.0) [39], and expression levels were reported as FPKM (Fragments Per Kilobase of transcript per Million mapped reads) to normalize for sequencing depth and transcript length effects. Differential expression analysis of transcriptome raw count data was performed using the DESeq2 R package (version 1.38.3) [40]. Differentially expressed genes (DEGs) were identified by applying a fold-change threshold of |log2FoldChange| ≥ 1 and controlling the false discovery rate (FDR < 0.01) using the Benjamini–Hochberg method, with a final significance threshold of adjusted *p*-value (padj) ≤ 0.05. DEG analysis between SQ and 8YQ was performed using tools available on the Omicshare platform [41]. For FOXO-related genes, we generated a gene expression heatmap. Expression heatmaps for different age groups were generated using an in-house R script with the ComplexHeatmap R package (v2.14.0) [42]. Raw counts were standardized by row-wise Z-scores before visualization. The R script is provided in Supplementary Data S1.

### 2.5. Molecular Identification of SODs in O. formosanus

To identify *SOD* gene candidates, a BLASTP search (e-value ≤ 1 × 10^−5^) was conducted against the transcriptome-predicted CDS translation protein library using all SOD protein sequences from the related termite *Zootermopsis nevadensis* as queries [43]. Following this initial screening, structural domains of all protein sequences were annotated using the InterProScan website [44], allowing for the preliminary identification of *SOD2* candidates based on the presence of both Cu/Zn and Fe/Mn binding domains. SignalP6.0 and TargetP2.0 were used to predict and localize signal peptides in all *SOD* sequences [45,46], aiding in the differentiation of *SOD1* and *SOD3* isoforms. Multiple sequence alignment was performed using the COBALT tool on NCBI “https://www.ncbi.nlm.nih.gov/tools/cobalt/re_cobalt.cgi (accessed on 25 August 2025)” [47], and results were visualized using Jalview (version 2.11.5.0) [48].

### 2.6. Quantitative Analysis of Candidate Genes

To validate the transcriptome results, quantitative real-time PCR (qRT-PCR) was performed on the remaining RNA samples. First-strand cDNA was synthesized using the HiScript IV 1st Strand cDNA Synthesis Kit (Vazyme Biotech Co., Ltd., Nanjing, China). The cDNA was stored at −20 °C for future use. Eleven genes were selected for qRT-PCR validation, with primer sequences provided in Table S1. Β-actin was used as the internal reference gene. Fluorescence quantification was performed using ChamQ SYBR qPCR Master Mix (Vazyme Biotech Co., Ltd., Nanjing, China) on a CFX96 Touch system (Bio Rad, Hercules, CA, USA). Thermal cycling conditions were as follows: 95 °C for 30 s, followed by 39 cycles of 95 °C for 10 s, and 60 °C for 10 s. Relative gene expression levels were calculated using the 2^−△Ct^ method [49]. Statistical analysis was conducted in R, where standard error (SE) and one-way ANOVA with Tukey’s HSD post hoc test were used to compare expression levels among groups. Results were visualized using the ComplexHeatmap R package, with significance represented in the compact letter display (CLD) format: identical letters indicate no significant difference, while different letters denote significant differences (*p* < 0.05).

### 2.7. SOD Enzyme Activity Assay

As a downstream effector protein, SOD exhibits functional relevance that is more broadly reflected by its enzyme activity at the whole-body level; therefore, whole-body samples were used for the enzyme activity assays. Total protein was extracted using the RIPA lysis method. SOD activity was quantified by generating O^2−^ through a xanthine–xanthine oxidase reaction system. The produced O^2−^ oxidizes hydroxylamine to form nitrite, which, after color development with a chromogenic reagent, was measured at 562 nm using an enzyme-linked immunosorbent assay (ELISA) plate reader. To differentiate between SOD isoforms based on metal cofactors, potassium cyanide (KCN) was added to selectively inhibit Mn-SOD activity. Subtracting Cu/Zn-SOD activity from total SOD activity provides Mn-SOD activity [50]. Three independent biological replicates from distinct colonies were conducted to ensure the reliability of the measurements for *O. formosanus* reproductives. All enzyme activity results were normalized to protein content. Protein concentration was quantified using the BCA assay after constructing a standard curve, which is shown in Figure S4.

## 3. Results

### 3.1. De Novo RNA-Seq Assembly

After primer removal, a total of 4,978,742 full-length non-concatemer reads were obtained, with read lengths ranging from 250 to 2500 bp (Figure S1). The clustering step yielded 38,438 unigenes with an average length of 1438 bp. After filtering adapters and low-quality reads, 4.97 million full-length cDNA fragments (average length: 1137 bp) were retained from the raw data. The IsoSec3 clustering process resulted in 300,115 cluster centers, with length distributions consistent with the initial set (Figure S2). A subsequent redundancy removal step using CD-HIT generated 38,483 high-quality unigenes (Figure S3).

### 3.2. Unigene Functional Annotation

**Figure 1:**
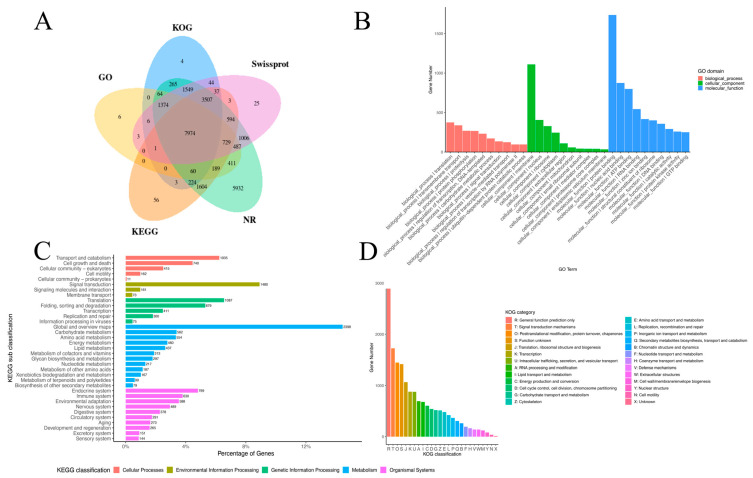
Functional annotation of the transcriptome reference from an 8YQ individual showing a Venn diagram of database matching (**A**) and histogram presentations of GO (**B**), KEGG (**C**) and KOG (**D**) classifications. A total of 15,112 unigenes were grouped into 26 KOG classifications. The bar heights/lengths indicate the number of unigenes in each specific functional cluster. Color legends represent functional categories.

Functional annotation was performed by searching against multiple databases, resulting in a total of 26,157 annotated unigenes (Figure 1A). In the Molecular Function category, 26,187 unigenes were annotated, with the most common terms being protein binding (1736 unigenes), nucleotide binding (875 unigenes), and ATP binding (799 unigenes). In the Biological Process category of GO, 24,450 unigenes were annotated, with frequent terms including translation (375 unigenes), transmembrane transport (338 unigenes), and proteolysis (269 unigenes). In the Cellular Component category, 24,073 unigenes were annotated, primarily in the membrane (1110 unigenes), nucleus (406 unigenes), and ribosome (328 unigenes) (Figure 1B). KEGG annotations were categorized into five major groups (Figure 1C): Cellular Processes (2363 unigenes), Environmental Information Processes (1714 unigenes), Genetics Information Processes (2752 unigenes), Metabolism (5770 unigenes), and Organismal Systems (4008 unigenes). Within the Metabolism category, the “Global and Overview maps” subcategory had the highest number of annotations (2398 unigenes), while the Endocrine system was the most annotated subcategory in Cellular Processes (799 unigenes). In the Environmental Information category, Translation was the most annotated subcategory (1087 unigenes), and Signal transduction was the most annotated subcategory in Environmental Information Processes (1480 unigenes). Additionally, Transport and catabolism were the most annotated subcategories in Cellular Processes (1035 unigenes) (Figure 1C). The top three KOG functional classifications were General function prediction only (3095 unigenes), Signal transduction mechanisms (1725 unigenes), and Posttranslational modification, protein turnover, and chaperones (1446 unigenes) (Figure 1D).

### 3.3. Differential Expression Analysis

**Figure 2:**
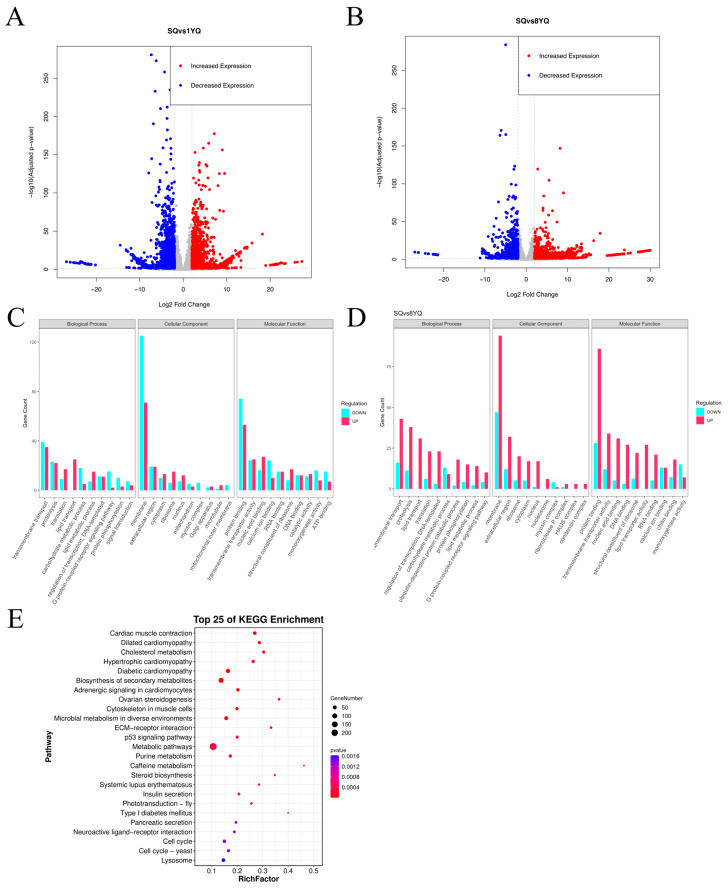
Volcano plots showing differentially expressed genes in SQ vs. 1YQ (**A**) and SQ vs. 8YQ (**B**). Histograms displaying GO annotation results of the differentially expressed genes in SQ vs. 1YQ (**C**) and SQ vs. 8YQ (**D**). Bubble plot showing the KEGG pathway enrichment of differentially expressed genes in SQ vs. 8YQ (**E**).

Comparisons between young (SQ vs. 1YQ) and old (SQ vs. 8YQ) termite queens revealed distinct gene expression patterns (Figure 2). The SQ vs. 1YQ comparison identified 4233 differentially expressed unigenes (2031 up-regulated and 2202 down-regulated), while the SQ vs. 8YQ comparison identified 3513 differentially expressed unigenes (2330 up-regulated and 1183 down-regulated) (Figure 2A,B,E). In the SQ vs. 1YQ comparison, 677 unigenes were annotated to Biological Processes, with transmembrane transport being the most represented subcategory (56 down-regulated and 42 up-regulated). In the Cellular Components category, 427 unigenes were annotated, with the membrane as the most abundant subcategory (129 down-regulated and 73 up-regulated). Among 498 unigenes annotated to Molecular Functions, protein binding was the most prevalent subcategory (67 down-regulated and 62 up-regulated) (Figure 2C). In the SQ vs. 8YQ comparison, 596 unigenes were annotated to Biological Processes, again with transmembrane transport as the most represented subcategory (16 down-regulated and 47 up-regulated). In the Cellular Components category, 258 unigenes were annotated, with the membrane as the most abundant subcategory (49 down-regulated and 96 up-regulated). Among 637 unigenes annotated to Molecular Functions, protein binding was the most prevalent subcategory (26 down-regulated and 102 up-regulated) (Figure 2D).

To identify key regulatory genes contributing to lifespan extension in elderly queens, KEGG pathway enrichment analysis was performed on the DEGs from the SQ vs. 8YQ comparison. The most significantly enriched pathway was the p53 signaling pathway, a central regulator involved in cell cycle control and DNA repair. Among the enriched KEGG pathways, insulin secretion was identified as one of the pathways potentially relevant to the present study. Because KEGG pathway annotations are largely derived from model organisms, including human-centered pathway definitions, the enriched term “insulin secretion” should be interpreted as a functionally related annotation rather than direct evidence of an identical physiological process in termites (Figure 2E).

**Figure 3:**
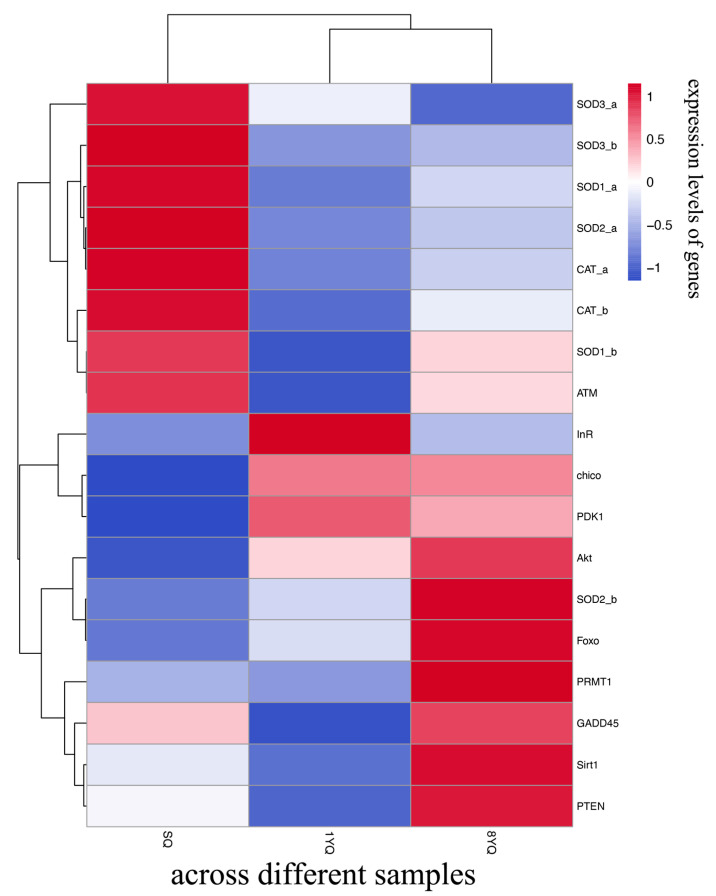
Hierarchical clustering heatmap showing the z-score-transformed expression levels of genes in the IIS pathway and FoxO-related downstream effector genes across different samples. The color scale indicates relative expression levels after row-wise z-score transformation.

**Figure 4:**
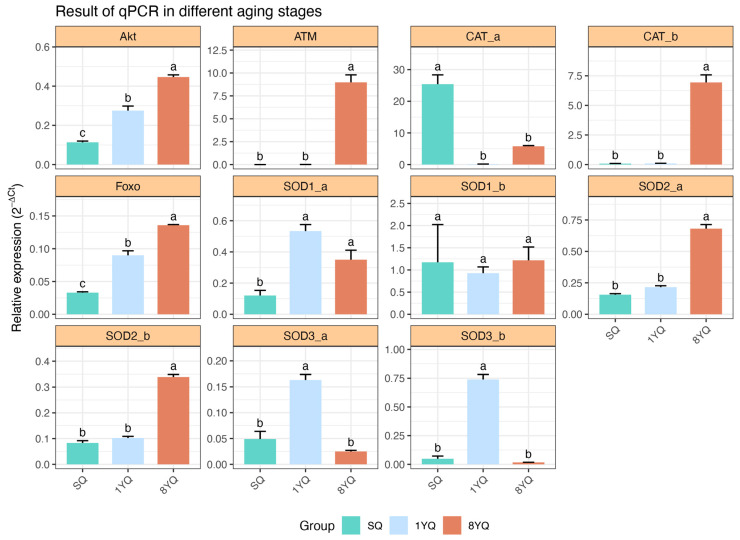
RT-qPCR results of selected IIS pathway genes and antioxidant genes in queen *O. formosanus* at different ages. The gene names are labeled above each individual histogram. Significance levels are indicated using the compact letter display (CLD) method. Different letters above the bars indicate significant differences among groups based on one-way ANOVA followed by Tukey’s HSD test (*p* < 0.05).

Given the link between these pathways and upstream IIS, the subsequent analysis focused on this pathway. RNA-seq expression levels of 11 downstream genes, including insulin receptor (*InR*), insulin receptor substrate (*chico*), *PDK*, *Akt*, *Sirt1*, and *FOXO*, revealed a gradual increase in expression with age. Additionally, the antioxidant gene *SOD2_b* (regulated by *FOXO*) and DNA repair-related genes *ATM* and *GADD45* also showed age-dependent upregulation (Figure 3). To validate the RNA-seq results, qRT-PCR was performed on these genes, and the expression patterns were consistent with the RNA-seq findings (Figure 4).

### 3.4. Identification and Characterization of SOD Transcripts in O. formosanus

**Figure 5:**
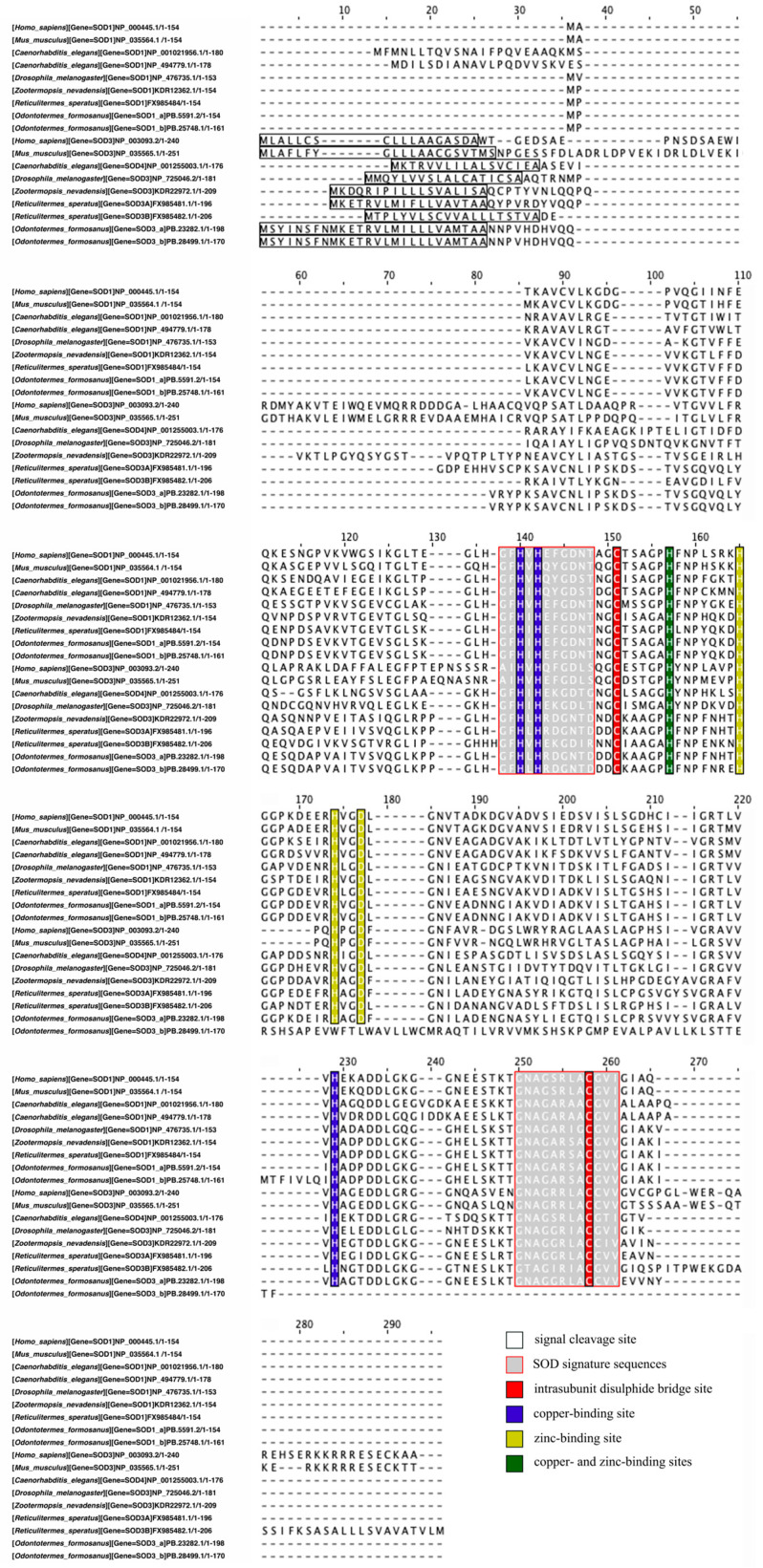
Multiple sequence alignment of SOD sequences from *Homo sapiens*, *Mus musculus*, *Caenorhabditis elegans*, *Drosophila melanogaster*, *Zootermopsis nevadensis*, *Reticulitermes speratus*, and *O. formosanus*.

Six *SOD* transcript candidates were identified in *O. formosanus*, classified into three SOD families (*SOD1*, *SOD2*, and *SOD3*) based on structural and functional characteristics. Four candidate *SOD1* transcripts were initially identified: PB.17935.1, PB.25748.1, PB.5591.1, and PB.23282.1. However, PB.17935.1 was excluded because it was annotated with an HMA/heavy-metal-associated domain and explicitly identified as “Copper chaperone for superoxide dismutase,” indicating that it is more likely a copper chaperone protein rather than a functional *SOD* transcript (Table S2). Three candidate *SOD2* transcripts were obtained: PB.3231.1, PB.22867.1, and PB.10913.1. PB.22867.1 was excluded because it lacked the conserved C-terminal Fe/Mn SOD domain required for a complete *SOD2* architecture, suggesting that it may not possess functional *SOD2* activity (Table S3). Five candidate *SOD3* transcripts were identified: PB.17935.1, PB.25748.1, PB.28499.1, PB.23282.1, and PB.5591.1. PB.17935.1 was excluded for the same reason (Table S4). Signal peptide prediction revealed distinct subcellular localizations: PB.5591.2 and PB.25748.1 were predicted as non-secreted proteins (cytoplasmic or nuclear), while PB.23282.1 and PB.28499.1 were identified as secreted proteins. PB.3231.1 and PB.10913.1 were predicted as mitochondrial proteins (Table 1). Multiple sequence alignment confirmed conserved Cu/Zn binding sites in PB.5591.2, PB.25748.1, PB.23282.1, and PB.28499.1, with PB.23282.1 and PB.28499.1 containing signal peptide fragments (Figure 5). Based on these analyses, two *SOD1* transcripts, two *SOD2* transcripts, and two *SOD3* transcripts were identified and named in *O. formosanus*: *SOD1_a* (PB.5591.1), *SOD1_b* (PB.25748.1), *SOD2_a* (PB.3231.1), *SOD2_b* (PB.10913.1), *SOD3_a* (PB.23282.1) and *SOD3_b* (PB.28499.1).

### 3.5. Analysis of SOD Enzyme Activity Across Different Reproductive Ages in O. formosanus

**Figure 6:**
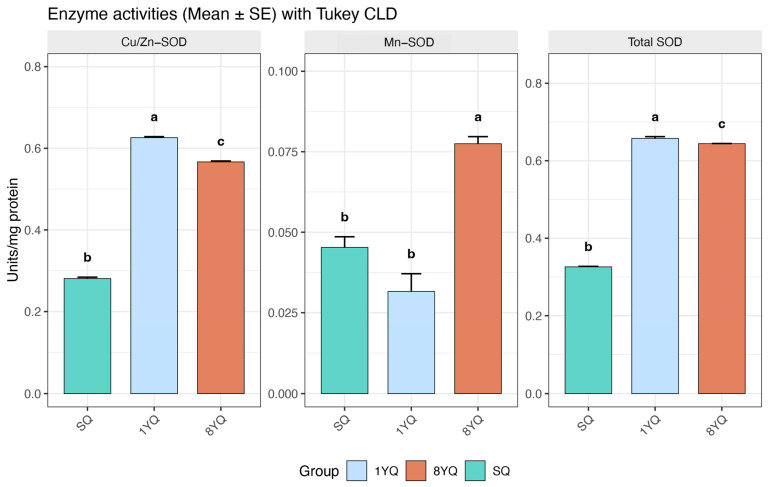
Histograms of SOD activity across ages of the *O. formosanus* queens. Different letters above the bars indicate significant differences among groups based on one-way ANOVA followed by Tukey’s HSD test (*p* < 0.05), using the compact letter display (CLD) method.

Analysis revealed distinct patterns of SOD enzyme activity across different reproductive stages in *O. formosanus* (Figure 6). Overall, SOD activity was highest in the YQ (young queen) group (0.6576 Units/mg protein), followed by the 8YQ group (0.6441 Units/mg protein), and lowest in the SQ group (0.3261 Units/mg protein). Cu/Zn-SOD was the predominant isoform, with activity peaking in YQ (0.6260 Units/mg protein), followed by 8YQ (0.5666 Units/mg protein), and lowest in SQ (0.2809 Units/mg protein). This suggests that Cu/Zn-SOD plays a dominant role in the antioxidant defense of young queens. In contrast, Mn-SOD activity was highest in the 8YQ group (0.0775 Units/mg protein), followed by SQ (0.0453 Units/mg protein), and lowest in YQ (0.0316 Units/mg protein). This indicates that Mn-SOD activity increases with reproductive age, potentially compensating for age-related oxidative stress.

## 4. Discussion

The IIS pathway, a central regulator of lifespan, plays a critical role in extending longevity across various model organisms. Initially identified in nematodes, the absence of its InR (*daf-2*) or downstream transcription factor FOXO (daf-16) significantly prolongs lifespan [20]. Similar lifespan-extending effects have been observed in mice and fruit flies, where inhibition of IIS increases longevity [19,51].

This pathway has been extensively studied in hymenopteran social insects [52], where it not only regulates reproductive rates in bees but also contributes to lifespan extension. In ants, the extended lifespan of gamergates is associated with reduced signaling activity in the downstream AKT/FOXO branch of the IIS pathway, consistent with their extended longevity [53]. In cockroaches, as reproductive lifespan increases, insulin activity also rises [54], supporting our findings. However, unlike in Hymenoptera, the IIS pathway in cockroaches does not undergo significant reorganization.

FOXO, a key transcription factor downstream of IIS, acts as a hub linking this pathway to several effector processes, including DNA repair, inflammation suppression, cell cycle regulation, antioxidant responses, and autophagy [22]. In most organisms, the IIS pathway activates InR substrates through InR activation, triggering the PI3K/Akt pathway. This leads to FOXO phosphorylation and the subsequent loss of its nuclear transcriptional activity [55].

**Figure 7:**
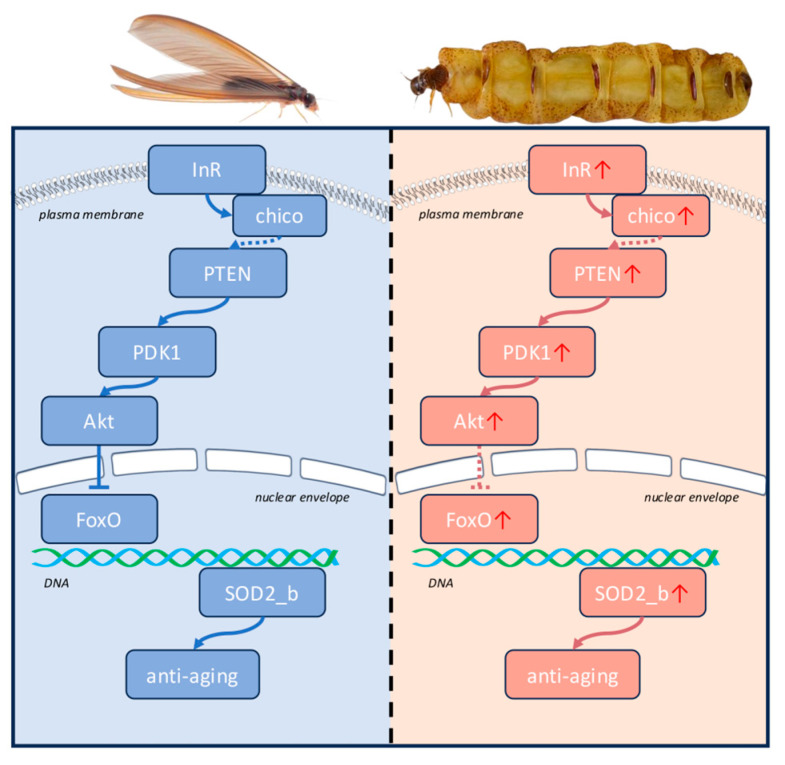
Schematic diagram of the molecular mechanisms underlying lifespan extension in *O. formosanus* queens. Arrowheads indicate activation, while flat-bottomed lines indicate inhibition. Solid lines represent direct regulatory interactions, and dashed lines represent indirect or putative regulatory interactions. The red arrows following the gene names indicate upregulation.

Transcriptome analysis revealed a gradual increase in the expression levels of *PDK1*, *Akt*, and *FOXO* in *O. formosanus* with age. The elevated expression of FOXO may mitigate the negative impact of the upstream IIS pathway on lifespan extension through compensatory effects (Figure 7). Additionally, the expression of Protein Arginine Methyltransferase 1 (PRMT1) was upregulated with age. This protein, regulated by ROS and mTOR signaling pathways, inhibits FOXO phosphorylation by p-Akt, stabilizing FOXO’s protein structure and maintaining its transcriptional activity [56]. This mechanism likely contributes to lifespan extension in *O. formosanus* by bypassing the IIS pathway.

Interestingly, in hymenopteran social insects, queens of ants and bees enhance FOXO’s transcriptional activity through deacetylation, counteracting the inhibitory effect of IIS on lifespan extension [53]. This finding offers a new perspective on lifespan regulation mechanisms across different social insect species.

FOXO transcription factors are central to lifespan extension by regulating various downstream biological processes. Transcriptome and RT-qPCR data confirm this, showing a significant increase in the expression of DNA repair-related genes (*ATM*, *GADD45*) and antioxidant genes (*SOD2*) in long-lived queens. In the antioxidant gene network regulated by *FOXO*, *SOD* plays a pivotal role in converting O^2−^ into hydrogen peroxide and oxygen molecules. This is followed by the metabolism of hydrogen peroxide into water (H_2_O) by catalase (CAT) or glutathione peroxidase (GPx) [26].

This study identified two subtypes of each of the three SOD genes in *O. formosanus*, whereas *SOD2* in a related termite exists as a single subtype [11]. This discrepancy may be associated with the demand for mitochondrial antioxidant regulation in *O. formosanus* during later life stages, but it cannot be excluded that it is partly attributable to technical biases arising from differences in transcriptome assembly, transcript reconstruction, and annotation criteria among studies. Therefore, it cannot yet be directly interpreted as biologically meaningful divergence with clear functional significance. The transcriptome and RT-qPCR results support this hypothesis, showing that, as the reproductive age of *O. formosanus* increases, the expression of *SOD2*, particularly *SOD2_b*, rises significantly.

The enhancement of SOD activity, primarily driven by SOD2, rather than SOD1 or SOD3, is attributed to the increased transcriptional activity of FOXO in older queens.

*SOD2* is a direct target gene of *FOXO*, whereas *SOD1* and *SOD3* are not. This observation indirectly supports our hypothesis that, in *O. formosanus*, reproduction may effectively counteract the potential lifespan-shortening effects of upstream IIS activation through a compensatory FOXO mechanism, thus maintaining robust downstream lifespan extension pathways.

The peak in total SOD activity in 1YQ, predominantly contributed by Cu/Zn SOD, can be partially attributed to the elevated expression of *SOD1_a*, *SOD3_3*, and *SOD3_b* in 1YQ and may also be influenced by age-related variations in Cu^2+^ concentration [57]. Significantly higher Cu^2+^ levels in reproductives compared to workers in a related termite [11] support this hypothesis. Recent work in *A. mellifera* suggests that reproductives undergo a reorganization of tissue- and caste-specific mitochondrial regulatory patterns reducing oxidative damage and contributing to their extended lifespan [58]. This observation may indicate that mitigating mitochondrial aging is a shared strategy among eusocial insects.

However, this study has several limitations. First, obtaining samples of older *O. formosanus* queens was challenging, with the oldest subjects being only 8 years old. This limits the ability to verify whether IIS in the queen continues to increase with age, whether FOXO transcription factor activity persists, whether SOD enzyme activity remains elevated or declines in later life stages, and whether the IIS–FOXO–SOD2 axis continues to function as an anti-aging mechanism throughout the aging process of reproductives. Second, the lack of direct experimental evidence on mitochondrial oxidative stress levels leaves our conclusions speculative, and we cannot conclusively determine whether the increased enzymatic activity of SOD2 truly alleviates mitochondrial oxidative stress. Further investigation of the underlying mechanisms is therefore required. In addition, this study did not include comparisons with male reproductives or worker castes. Based on previous studies in *R*. *chinensis* and *R*. *speratus*, young queens in *R. chinensis* exhibit lower IIS expression than workers, whereas in *R. speratus*, the Mn-SOD enzymatic activity of queens does not differ significantly from that of workers [3,11]. Therefore, we are currently unable to determine whether the anti-aging mechanism centered on the IIS–FOXO–SOD2 axis in *O. formosanus* queens is queen-specific or unique to *O. formosanus*. Most importantly, the regulatory relationship between FOXO and SOD2 remains inadequately understood, which will be a primary focus of future research.

## Figures and Tables

**Table 1:** Predicted location and signal cleavage site of putative SOD proteins.

Gene Sequence (Database Number)	Localization (Reliability Class)	Signal Cleavage
NP_000445.1 [Gene=SOD1] [*Homo sapiens*]	-	None
NP_035564.1 [Gene=SOD1] [*Mus musculus*]	-	None
NP_001021956.1 [Gene=SOD1] [*Caenorhabditis elegans*]	-	None
NP_494779.1 [Gene=SOD1] [*Caenorhabditis elegans*]	-	None
NP_476735.1 [Gene=SOD1] [*Drosophila melanogaster*]	-	None
KDR12362.1 [Gene=SOD1] [*Zootermopsis nevadensis*]	-	None
FX985484 [Gene=SOD1] [*Reticulitermes speratus*]	-	None
PB.5591.2 [Gene=SOD1_a] [*Odontotermes formosanus*]	-	None
PB.25748.1 [Gene=SOD1_b] [*Odontotermes formosanus*]	-	None
NP_003093.2 [Gene=SOD3] [*Homo sapiens*]	Secreted (Likelihood = 0.9991)	1–18
NP_035565.1 [Gene=SOD3] [*Mus musculus*]	Secreted (Likelihood = 1)	1–20
NP_001255003.1 [Gene=SOD4] [*Caenorhabditis elegans*]	Secreted (Likelihood = 0.9988)	1–17
NP_725046.2 [Gene=SOD3] [*Drosophila melanogaster*]	Secreted (Likelihood = 0.9992)	1–18
KDR22972.1 [Gene=SOD3] [*Zootermopsis nevadensis*]	Secreted (Likelihood = 0.9991)	1–18
FX985481.1 [Gene=SOD3A] [*Reticulitermes speratus*]	Secreted (Likelihood = 0.9992)	1–18
FX985482.1 [Gene=SOD3B] [*Reticulitermes speratus*]	Secreted (Likelihood = 0.9991)	1–20
PB.23282.1 [Gene=SOD3_a] [*Odontotermes formosanus*]	Secreted (Likelihood = 0.9992)	1–26
PB.28499.1 [Gene=SOD3_b] [*Odontotermes formosanus*]	Secreted (Likelihood = 0.9992)	1–26
NP_001019636.1 [Gene=SOD2] [*Homo sapiens*]	Mitochondrial (Likelihood = 0.969)	1–24
NP_038699.2 [Gene=SOD2] [*Mus musculus*]	Mitochondrial (Likelihood = 0.9532)	1–24
NP_492290.1 [Gene=SOD2] [*Caenorhabditis elegans*]	Mitochondrial (Likelihood = 0.7115)	1–24
NP_510764.1 [Gene=SOD3] [*Caenorhabditis elegans*]	Mitochondrial (Likelihood = 0.9632)	1–24
NP_476925.1 [Gene=SOD2] [*Drosophila melanogaster*]	Mitochondrial (Likelihood = 0.9252)	1–17
KDR21306.1 [Gene=SOD2] [*Zootermopsis nevadensis*]	Secreted (Likelihood = 0.941989)	1–80
FX985482.1 [Gene=SOD2] [*Reticulitermes speratus*]	Other (Likelihood = 0.852)	20–30
PB.3231.1 [Gene=SOD2_a] [*Odontotermes formosanus*]	Mitochondrial (Likelihood = 0.6184)	20–30
PB.10913.1 [Gene=SOD2_b] [*Odontotermes formosanus*]	Mitochondrial (Likelihood = 0.5958)	23–176

## Data Availability

The original contributions presented in this study are included in the article/Supplementary Material. Further inquiries can be directed to the corresponding author.

## Supplementary Materials

The following supporting information can be downloaded at: https://www.mdpi.com/article/10.3390/insects17040432/s1, Figure S1. Length distribution of full-length isoform reads after lima and refine processing. Figure S2. Length distribution of clustered and polished isoform consensus sequences generated by IsoSeq3. Figure S3. Length distribution of final non-redundant unigenes after CD-HIT clustering. Figure S4. Standard curve. Table S1. Primer sequences used for RT-qPCR of the selected 11 genes, with β-actin as the internal reference. Table S2. Candidate SOD1 genes identified by BLASTP and InterProScan domain annotation, with exclusion of putative pseudogenes. Table S3. Candidate SOD2 genes identified by BLASTP and InterProScan domain annotation, with exclusion of sequences containing premature stop codons. Table S4. Candidate SOD3 genes identified by BLASTP and InterProScan domain annotation, with exclusion of putative pseudogenes. Data S1: The R script for Figures S1–S4.
