# Supplementary figures and images for: Transcriptomic Evidence Reveals the IIS–FOXO–SOD2 Axis as a Core Anti-Aging Pathway in Long-Lived Queens of Odontotermes formosanus
Source: Insects. 2026 Apr 17;17(4):432. doi: 10.3390/insects17040432 (PMC13116683; doi:10.3390/insects17040432)

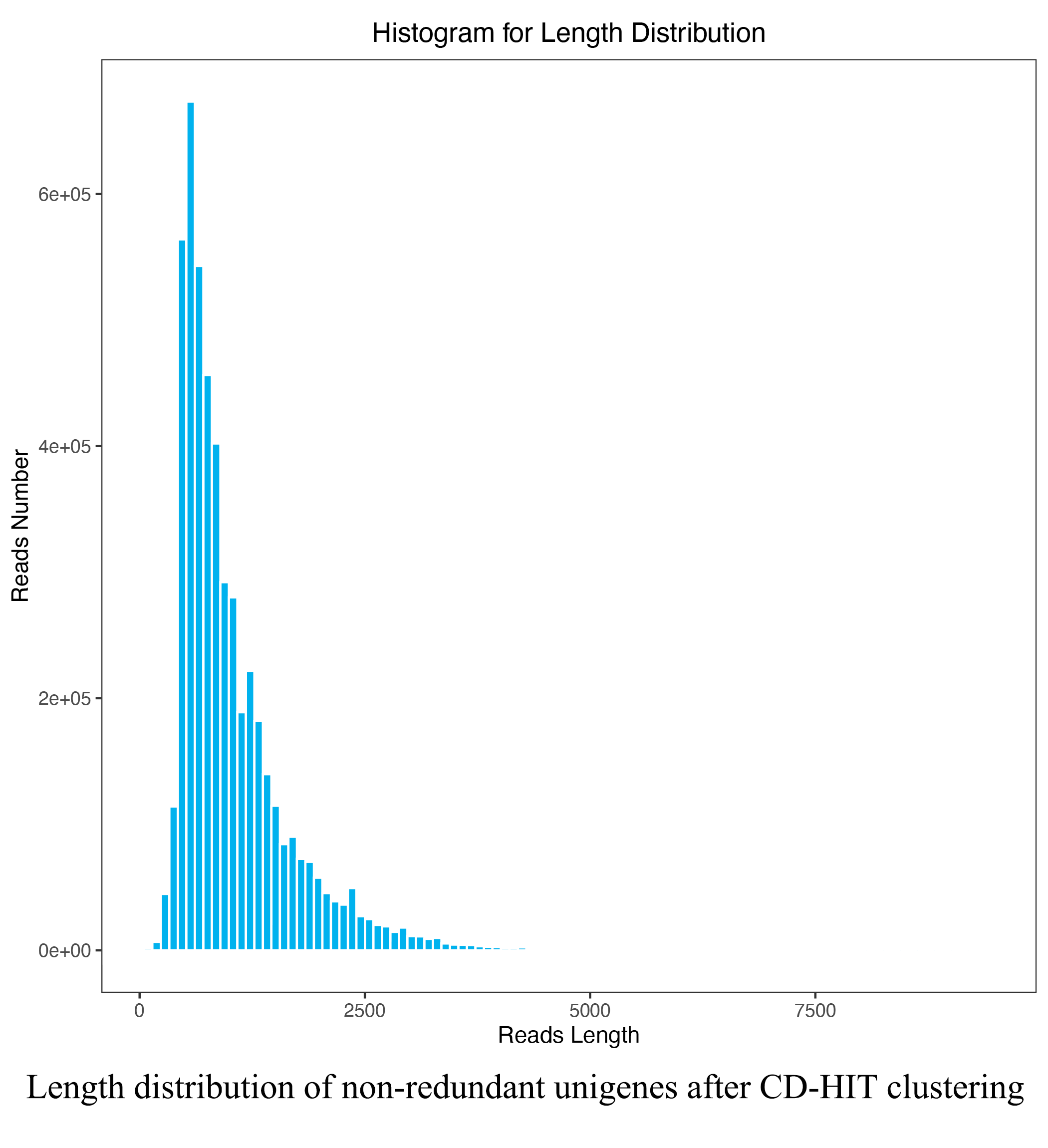

Supplement: Supplementary file 1 [file insects-17-00432-s001.zip › Figure S1. Length distribution of full-length isoform reads after lima and refine processing.png]

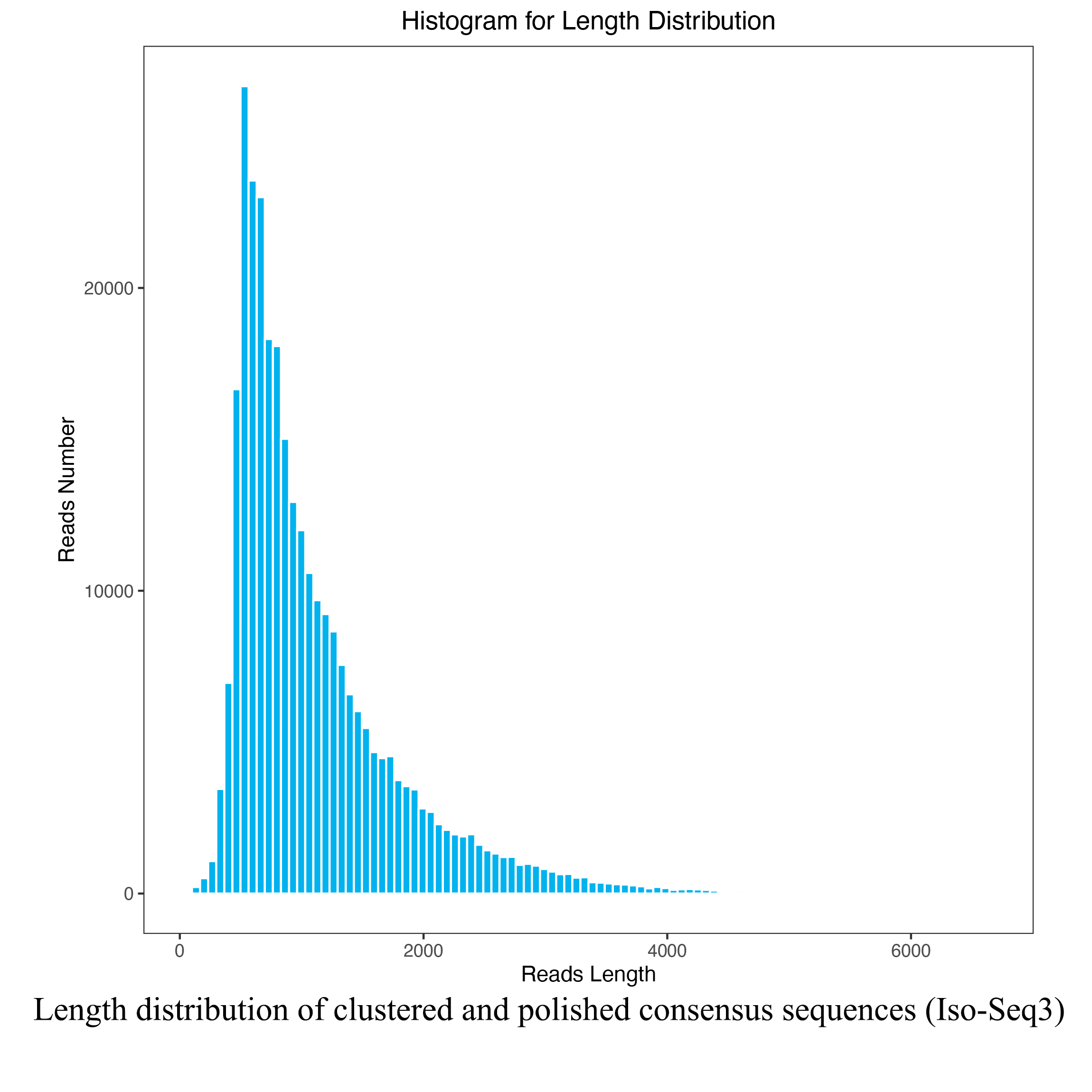

Supplement: Supplementary file 1 [file insects-17-00432-s001.zip › Figure S2. Length distribution of clustered and polished isoform consensus sequences generated by IsoSeq3.png]

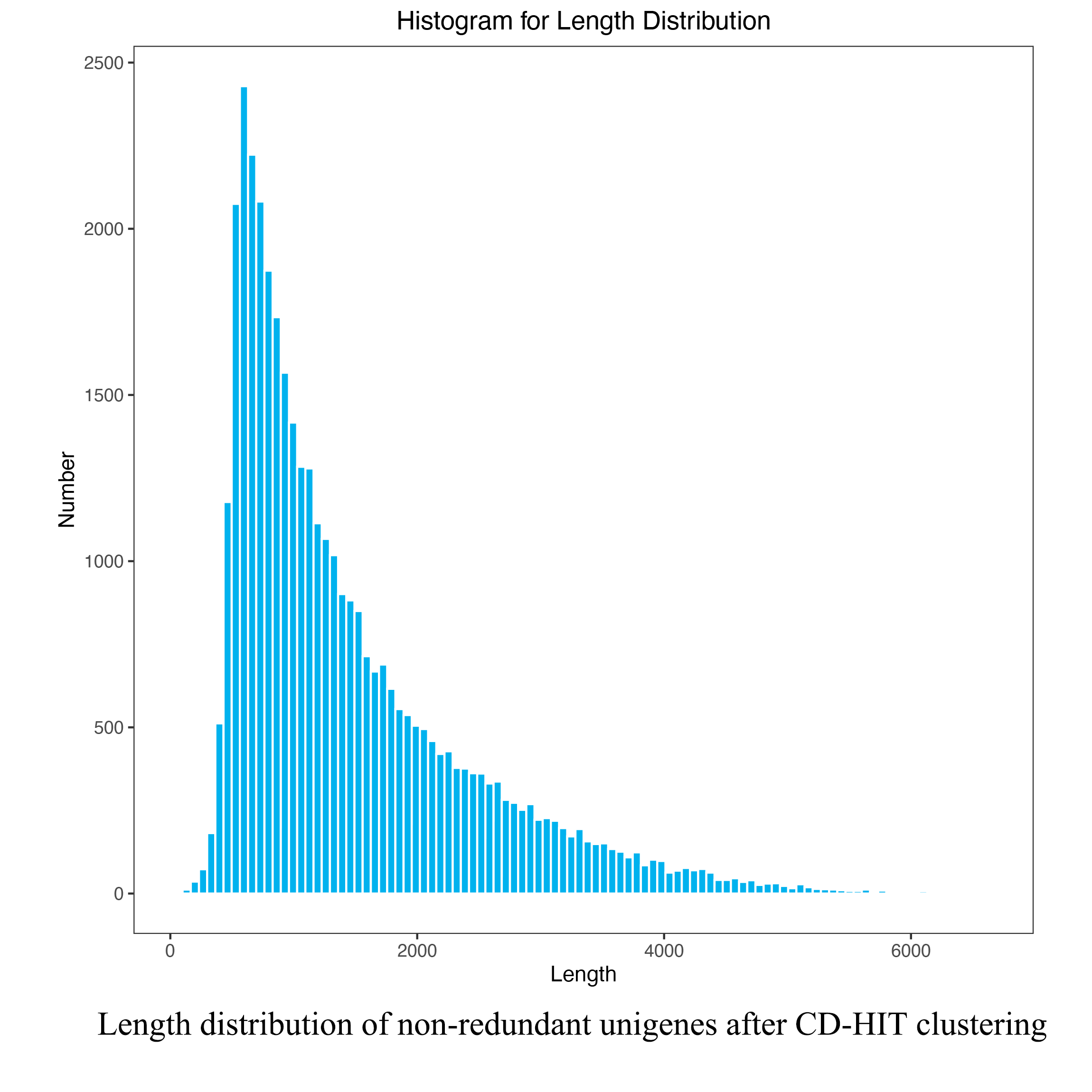

Supplement: Supplementary file 1 [file insects-17-00432-s001.zip › Figure S3. Length distribution of final non-redundant unigenes after CD-HIT clustering.png]

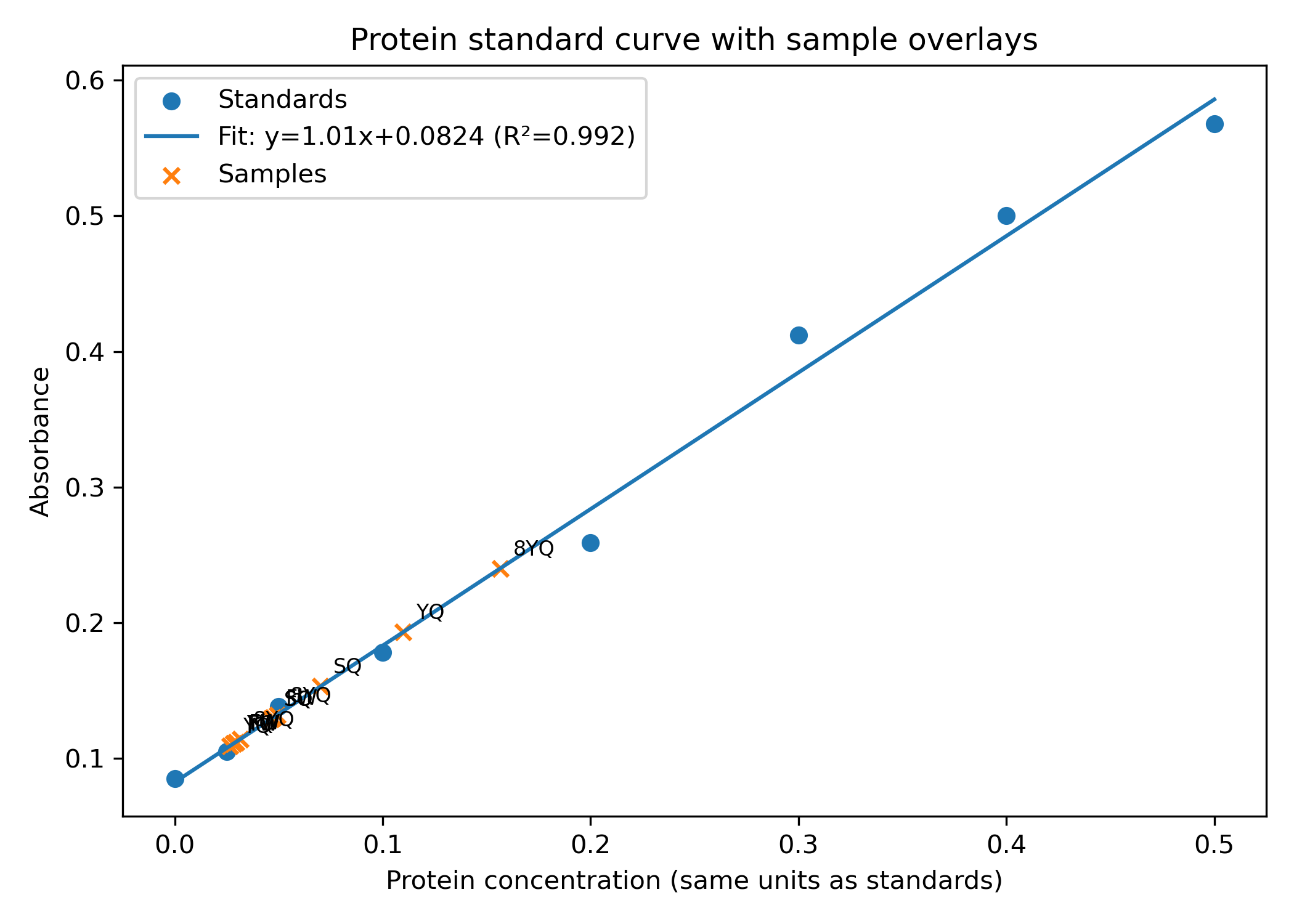

Supplement: Supplementary file 1 [file insects-17-00432-s001.zip › Figure S4.standard curve.png]
